# Changes in Blood Metabolic Profiles Reveal the Dietary Deficiencies of Specific Nutrients and Physiological Status of Grazing Yaks during the Cold Season in Qinghai Province of China

**DOI:** 10.3390/metabo12080738

**Published:** 2022-08-11

**Authors:** Jian Gao, Deyu Yang, Zhanying Sun, Jianzhang Niu, Yuhong Bao, Suozhu Liu, Zhankun Tan, Lizhuang Hao, Yanfen Cheng, Shujie Liu

**Affiliations:** 1 Laboratory of Gastrointestinal Microbiology, National Center for International Research on Animal Gut Nutrition, Nanjing Agricultural University, Nanjing 210095, China; 2 Key Laboratory of Plateau Grazing Animal Nutrition and Feed Science of Qinghai Province, Qinghai Plateau Yak Research Center, Qinghai Academy of Animal Science and Veterinary Medicine of Qinghai University, Xining 810016, China; 3 Institute of Grassland Science, Tibet Academy of Agricultural and Animal Husbandry Sciences, Lhasa 850000, China; 4 College of Animal Science, Tibet Agricultural and Animal Husbandry University, Nyingchi 860000, China

**Keywords:** blood metabolome, metabolic profile, nutrient deficiency, grazing yak

## Abstract

This study aimed to investigate the changes in the blood metabolic profiles of grazing yaks during the cold season to reveal their physiological status and seek the nutrients needed to be supplemented. Six castrated yaks (3 years old) with 166.8 kg (standard deviation = 5.3) of liveweight grazed in the Qinghai-Tibetan Plateau were used as experimental animals without supplementary feeding. Blood samples of each animal were collected in October and December 2015, and March 2016 for the analysis of serum biochemicals and metabolome. Results showed serum indices involved in protein metabolism in grazing yaks showed greater differences during the cold season than the metabolisms of energy or minerals. Cold stress in December had minor effects on the serum metabolic profiles of yaks compared with those in October. Yaks in October and December shared seven differential serum metabolites and enrichments of the “arachidonic acid metabolism” and “glycine, serine, and threonine metabolism” pathways compared with those in March caused by the shortage of feeds. Summarily, the nutrient deficiency would be influential on the physiological status of grazing yaks during the cold season, especially on the protein metabolism, which could be improved by supplementary feeds.

## 1. Introduction

Yaks (*Bos grunniens*) are special livestock inhabited at the Qinghai-Tibet Plateau in China. These animals are key economic incomes for the local herders for providing fur, meat, and milk. Although yaks can tolerate the harsh conditions of this ecosystem such as hypoxia, intense ultraviolet radiation, and feed deficiency [1], the breeding levels of yaks are still low. Most of them are fully grazed in the grassland of Qinghai-Tibet Plateau [2], because the harsh environment in this plateau could not meet the cultivation of common feed crops for the local livestock. Due to the feeding pattern, yaks suffer nutrient deficiencies and cold stress during the cold season, which directly affects their blood metabolic profiles and performances. Previous research reported that both above-ground biomasses and nutritive values (especially energy and nitrogen) of forages in the Qinghai-Tibet Plateau decreased hugely during the cold season [2,3]. Besides, yaks also suffer cold stress caused by the low temperature during the cold season in the highlands. Previous research reported that extreme cold conditions have profound effects on serum metabolic profiles [4] and productivity [5] in ruminants. Thus, it is not surprising that the shortage of feeds and the cold stress cause the liveweight loss of grazing yaks, even up to 25% of total liveweight without supplementary feeds during the cold season [6].

During long-term evolution, yaks could partly adapt to the harsh environment in the highlands. The characteristics and functions of the rumen microbiome in yaks changed by altitude [7,8] and seasonal dynamics [2,9] to adapt to the forage composition in the highlands. This adaptation contributed to no differences in the ruminal efficiency of microbial protein synthesis under the shortage of feed sources [10] and greater ruminal utilization of lignocellulosic plants compared with other domestic animals [11,12]. Furthermore, yaks have greater tolerance to cold stress than other ruminants. The longer and denser fur covered the body surface, and the smaller and fewer sweat glands in the skin of yaks would abate the harmful effects of cold stress during the cold season [13]. Unfortunately, the adaptation of yaks to the highlands mentioned above could not help them to maintain their liveweight during the cold season [6]. Researchers reported that supplementary feeds can attenuate the harmful effects of nutrient deficiencies on the grazing yaks [14]. However, of the nutrient ingredients, which one is the most important for grazing yaks is still unclear. Thus, it is difficult to avoid the liveweight losses and maintain the health status of grazing yaks by supplementary feeding precisely.

Blood parameters are important indicators of animal health. The study of hematological and biochemical parameters helps in understanding the relationship of blood characteristics to the habitat and the adaptability of the animals to their environment. Changes in blood parameters depend on the species, sex, age, and nutritional and physical condition of animals [15,16,17,18,19]. The investigation of blood parameters in animals represents a crucial tool in the advancement of management conditions, especially concerning the detection of healthy versus infected or stressed animals [20,21,22,23,24]. Blood metabolic profiles including the biochemicals, low-molecular metabolites, cations, and anions are the indicators of the metabolic status in ruminants [25]. Puppel and Kuczyńska [26] reviewed the changes in the blood metabolic profiles of perinatal cows and concluded that the increased concentrations of non-esterified fatty acid and β-hydroxybutyric acid (a ketone body) in the blood are the most commonly used indicators for the negative energy balance of hosts. Yaks showed similar results during starvation with lower concentrations of glucose and triglyceride and greater concentrations of ketone body and non-esterified fatty acid in their serum [27,28]. For the protein status of hosts, blood protein and urea nitrogen are important indices to indicate the protein intake and protein absorption of digestive tracts [29]. Cold stress also affects the carbohydrate metabolism and protein status in ruminants, which could reflect using the biochemical parameters of their blood [4]. The above biochemicals are useful for predicting the requirement of major nutrients of ruminants. Metabolomic approaches are powerful tools to investigate the low-molecule metabolites in biological fluids which could reflect the metabolic changes of animals precisely. Recent studies have investigated the energy and protein metabolism of yaks in response to supplementary feeds during the warm season [30] or the cold season [31] using metabolomics technologies. However, the dynamic changes in the blood metabolome of grazing yaks in response to the cold season are still unclear, which causes difficulties for supplementary nutrients precisely for these yaks.

Based on the above results, we hypothesize that cold stress and dietary deficiencies during the cold season affect the blood metabolic profiles of grazing yaks, which reflect the shortage of specific nutrients for these animals. The purpose of the current study was to investigate the changes in serum biochemical indices including minerals as well as the low-molecule metabolites identified by the untargeted metabolome in grazing yaks during the cold season. Results obtained in this study would help to improve the supplementary feed strategies and the precise nutrition for grazing yaks in the highlands with harsh conditions.

## 2. Materials and Methods

### 2.1. Experimental Design and Sampling

Six castrated yaks (3 years old) with an average liveweight of 166.8 kg (standard deviation = 5.3) were used as the experimental animals. This experiment contained 15 d for adaption and 150 d for the trial period from 11 October 2015 to 25 March 2016. Before the beginning of this experiment, yaks were kept free of ecto- and endo-parasites using albendazole. All animals were grazed in the same meadow by local farmers (101°4′46″ E, 37°4′46″ N, Haibei, Qinghai) from 07:00 to 18:00 h daily without any supplementary feeding and were given free access to fresh drinking water. The experimental site is 3160 m above sea level with an average annual temperature of 7.6 °C. During the experimental period, the average temperature was −3 °C.

Blood samples were collected from the jugular vein using the vacutainers for serum collection with coagulants (BD Medical Instrument Co., Ltd., Shanghai, China) and ruminal contents were sampled simultaneously through the esophagus using a tube from each yak on 25 October 2015 (Oct, −1 to 11 °C), 25 December 2015 (Dec, −24 to −4 °C), and 25 March 2016 (Mar, −12 to 4 °C) before grazing in the morning. Serum samples were achieved by centrifuging the blood samples at 2000× *g* for 10 min using the Pico-21 tabletop centrifuge of ThermoFisher Scientific (China) Co., Ltd. (Shanghai, China). Liveweight of the yaks was obtained using the weighing scale platform (0.5 kg accuracy) purchased from Zhengfeng Electronic Technology Ltd. (Suzhou, Jiangsu province, China). Meanwhile, the plant biomass of the experimental meadow was recorded and forage samples were collected in several areas of the meadow on the same sampling days. Forage samples were stored at −20 °C, while the samples of serum and ruminal content were stored at −80 °C for later analysis.

### 2.2. Analysis of Forage, Rumen Content, and Serum Indices

The forage samples were air-dried at 65 °C and ground for the analysis of nutrient composition. Dry matter, ether extract, and crude ash in forages were determined using the methods of AOAC [32]. The content of organic matter in forages was the difference between the contents of dry matter minus crude ash. The nitrogen content of forages was analyzed by the Kjeldahl method as described in no. 988.05 of AOAC [32] and crude protein was calculated by nitrogen% × 6.25. Fiber components of forages including neutral detergent fiber and acid detergent fiber were analyzed according to Van Soest et al. [33]. Contents of non-fiber carbohydrates in forages were calculated by the contents of dry matter minus the percentages of crude protein, ash, ether extract, and neutral detergent fiber. The contents of calcium and total phosphorus in forages were analyzed using the methods of AOAC [32]. Ruminal ammonia-N and microbial crude protein were analyzed using the methods of Broderick and Kang [34] and Makkar et al. [35], respectively.

The concentrations of serum biochemical indices were all analyzed using the Roche Cobas^®^ 8000 modular analyzer in Qinghai Provincial People’s Hospital and related kits purchased from Roche Diagnostics (Basel, Switzerland), including indices involved with energy metabolism (glucose, total cholesterol, triglyceride), protein metabolism (total protein, albumin, globulin, urea nitrogen), and metabolic enzymes (alanine transaminase, aspartate aminotransferase, alkaline phosphatase, lactate dehydrogenase). For the determination of mineral elements, serum samples were digested with concentrated HNO_3_ for a night and then heated at 80 °C for 5 h on a hot plate. Concentrations of mineral elements in the digested serum including K, Na, Ca, Mg, and Fe were determined using the inductively coupled plasma optical emission spectroscopy (iCAP, ThermoFisher Scientific (China) Co., Ltd., Shanghai, China) as per the method of Harrington et al. [36].

### 2.3. Pretreatment and Identification of Serum Metabolites by GC-TOF/MS

A 100 μL serum sample of each yak was fully mixed with 0.35 mL methanol and 20 μL L-2-chlorophenylalanin (purity ≥ 98%, Shanghai Hengbai Biotech Co., Ltd., Shanghai, China) aqueous solution (1 mg/mL) as the internal standard. After fully mixing, the mixture was then centrifuged at 13,000× *g* under 4 °C for 15 min using the 5425R microcentrifuge (Eppendorf, Hamburg, Germany). The supernatant was concentrated and incubated with methoxy amination hydrochloride (20 mg/mL in pyridine) at 80 °C for 30 min. Then, the solution was subsequently incubated with bis(trimethylsilyl)trifluoroacetamide containing 1% trimethylchlorosilane (*v*/*v*) (Regis Technologies, Inc., Morton Grove, IL, USA) at 70 °C for 2 h to conduct the pre-column derivatization of the serum metabolites. Untargeted serum metabolome was conducted using the Agilent 7890 gas chromatograph system coupled with a Pegasus HT time-of-flight mass spectrometer (GC-TOF/MS, LECO, St. Joseph, MI, USA). A DB-5MS capillary column (30 m × 250 μm inner diameter, 0.25 μm film thickness) coated with 5% diphenyl cross-linked with 95% dimethylpolysiloxane (J&W Scientific, Folsom, CA, USA) was used to separate the serum metabolites. Test conditions of the gas chromatograph and mass spectrometer were described in detail by Sun et al. [37].

### 2.4. Bioinformatic Analysis of Untargeted Serum Metabolome

Raw peaks were processed using Chroma TOF software (version 4.3X) and identified with the LECO-Fiehn Rtx5 database (LECO, St. Joseph, MI, USA) as the methods described by Kind et al. [38]. Peak areas of metabolites in each sample were normalized by the peak area of internal standard (L-2-chlorophenylalanin) and subsequently analyzed using MetaboAnalyst (version 5.0, https://www.metaboanalyst.ca, accessed on 13 April 2022) [39]. Metabolites were removed when they were not annotated or more than two-thirds of each sample were missing values. The method of k-nearest neighbor was used to estimate the missing values and data were normalized by the log transformation for later analysis. Chemometrics analysis of serum metabolites for all groups including the principal component analysis (PCA) and sparse partial least squares-discriminant analysis (PLS-DA) were conducted using MetaboAnalyst (version 5.0). The differential serum metabolites between every two groups were identified based on the variable importance in projection (VIP > 1) from orthogonal PLS-DA and the false discovery rate (FDR) from *t*-tests (FDR < 0.05). Fold changes were calculated by the area of each metabolite between groups (former month/later month), while pathway impact analysis was conducted with the peak area of differential serum metabolites using the MetaboAnalyst (version 5.0) and the Kyoto encyclopedia of genes and genomes (updated October 2019, https://www.genome.jp/kegg/, accessed on 13 April 2022) [40]. The enriched pathways with pathway impact > 0.1 and FDR < 0.05 were deemed as the key differential pathways between groups. The random forest analysis of differential serum indices or metabolites was conducted using the Wekemo Bioincloud (https://www.bioincloud.tech, accessed on 17 April 2022) and only the results with FDR < 0.05 were shown.

### 2.5. Statistical Analysis

Nutritional values of forage samples among different sampling days were analyzed by one-way analysis of variance, while serum biochemical indices were analyzed by the one-way analysis of covariance with the ID number of yaks as the covariant using SPSS software (version 26, IBM Corporation, Chicago, IL, USA). Multiple comparisons of Bonferroni correction were used to identify the difference between groups under the limitation of the family-wise error rate. All data were tested for normality before statistical analysis. The results were presented as mean ± standard deviation or mean ± pooled SEM. *p*-values or FDR < 0.05 were identified as the significant differences among groups.

## 3. Results

### 3.1. Nutritional Value of Forages, Yaks’ Liveweight, and Ruminal N Metabolism

**Figure 1:**
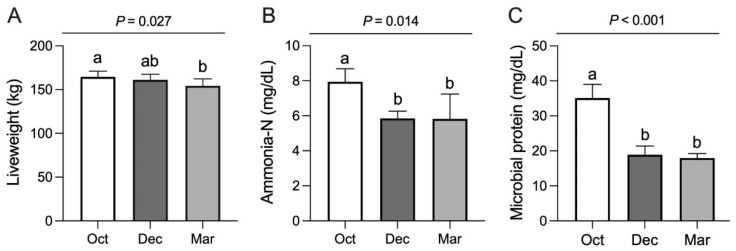
Dynamic changes in (**A**) liveweights, concentrations of (**B**) ammonia-N and (**C**) microbial crude protein in the ruminal fluid of grazing yaks during the cold season. Values with different letters represent significant differences among groups (*p* < 0.05). Results are presented as mean ± standard deviation.

During this experiment, the average temperatures in October, December, and March were −4 to 11 °C, −20 to 0 °C, and −9 to 6 °C, respectively. In October and December, the plant biomasses in the experimental meadow were 120.63 g/m^2^ and 124.53 g/m^2^ on an air-dried basis, while it was much lower in March (3.32 g/m^2^). The contents of organic matter and fibrous materials showed no difference in the forages collected in different months of the cold season (*p* > 0.10, Table 1). However, forages in October had greater content of crude protein than those collected in December (*p* = 0.010), while forages collected in March had lower content of ether extract than the other two months (*p* = 0.002). During the cold season, the liveweights of grazing yaks decreased significantly (*p* = 0.027) and ruminal fluid collected in October had greater concentrations of ammonia-N (*p* = 0.014) and microbial crude protein (*p* < 0.001) than those collected in the other two months (Figure 1).

### 3.2. Serum Biochemical Indices and Mineral Elements of Yaks

Table 2 shows that grazing yaks in October had the greatest serum concentrations of total protein (*p* < 0.001), globulin (*p* = 0.001), and activity of aspartate aminotransferase (*p* = 0.007), while they had the lowest total cholesterol in serum (*p* < 0.001) compared with those in the other two months. Significant differences were found in the serum albumin of yaks between December and March (*p* = 0.044), albumin to globulin ratio between October and December (*p* = 0.012), and activity of lactate dehydrogenase between October and March (*p* = 0.001). The grazing yaks in March had the lowest serum urea nitrogen compared with other months (*p* = 0.002). Furthermore, no differences were found in the serum concentrations of glucose, triglyceride, and the activities of alanine transaminase and alkaline phosphatase (*p* > 0.05, Table 2). For the serum minerals of grazing yaks during the cold season, yaks in October had greater serum sodium than in December (*p* = 0.038), greater magnesium than in December and March (*p* = 0.005), and tended to increase serum phosphorus compared with other sampling times (*p* = 0.053). Yaks in March had the lowest serum potassium than in other months (*p* < 0.001), while no differences were found in serum calcium and iron (*p* > 0.05, Table 2).

### 3.3. Serum Metabolome of Yaks

**Figure 2:**
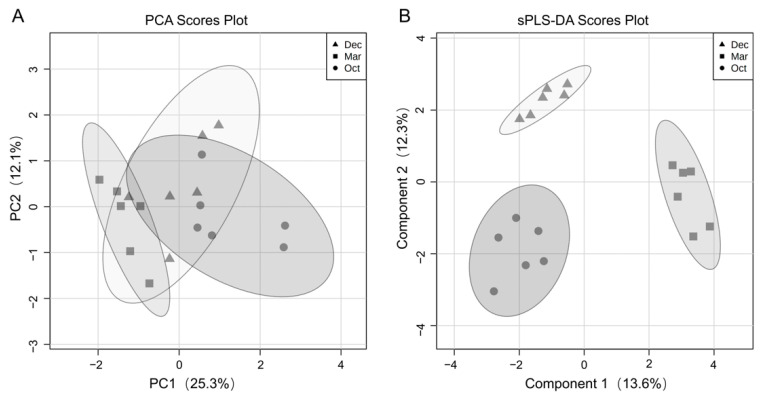
(**A**) Principal component analysis (PCA) and (**B**) sparse partial least squares-discriminant analysis (sPLS-DA) of serum metabolites in grazing yaks during the cold season.

Raw results of serum samples based on GC-TOF/MS obtained 436 valid peaks. After filtering, 393 peaks were kept and 163 metabolites were annotated effectively. Results of PCA based on GC-TOF/MS metabolic profiles showed no clear separation (Figure 2A), while results of sparse PLS-DA showed a clear separation with a 0.111 error rate among all groups (Figure 2B). Models of orthogonal PLS-DA were used for the analysis of differential between groups. Results showed that the corresponding R^2^Y and Q^2^ in the models between each two groups were R^2^Y = 0.913, Q^2^ = 0.699 (Oct vs. Dec), R^2^Y = 0.932, Q^2^ = 0.697 (Oct vs. Mar), R^2^Y = 0.962, Q^2^ = 0.646 (Dec vs. Mar), respectively, suggesting orthogonal PLS-DA models were effective for the identification of differential serum metabolites between each of the two groups.

A total of 17 differential metabolites were obtained in the serum samples based on the VIP obtained from orthogonal PLS-DA (VIP > 1) and the FDR from the student’s *t*-test (FDR < 0.05, Table 3). Of them, 5 (Oct vs. Dec), 9 (Oct vs. Mar), and 12 (Dec vs. Mar) differential metabolites were identified in the serum of yaks during the cold season, respectively. Compared with December, yaks in October had greater serum concentrations of lyxose (FDR = 0.019), threitol (FDR = 0.027), tyrosine (FDR = 0.027), leucrose (FDR = 0.035), and lower threonic acid (FDR = 0.019). Compared with March, yaks in October had greater serum threitol (FDR = 0.003) and lactic acid (FDR = 0.048), while they had lower serum monostearin (FDR *<* 0.001), 1-monopalmitin (FDR *<* 0.001), threonic acid (FDR *<* 0.001), D-(glycerol 1-phosphate) (FDR = 0.001), arachidonic acid (FDR = 0.004), glycine (FDR = 0.027), and gluconic acid (FDR = 0.033). All 12 differential metabolites of yak serum were lower in December compared with March, including monostearin (FDR *<* 0.001), 1-monopalmitin (FDR *<* 0.001), D-(glycerol 1-phosphate) (FDR *<* 0.001), arachidonic acid (FDR *<* 0.001), α-ketoglutaric acid (FDR = 0.011), gluconic acid (FDR = 0.012), serine (FDR = 0.019), glycine (FDR = 0.019), phosphate (FDR = 0.023), 3-hydroxybenzoic acid (FDR = 0.033), glucuronic acid (FDR = 0.034), and threonic acid (FDR = 0.047).

**Figure 3:**
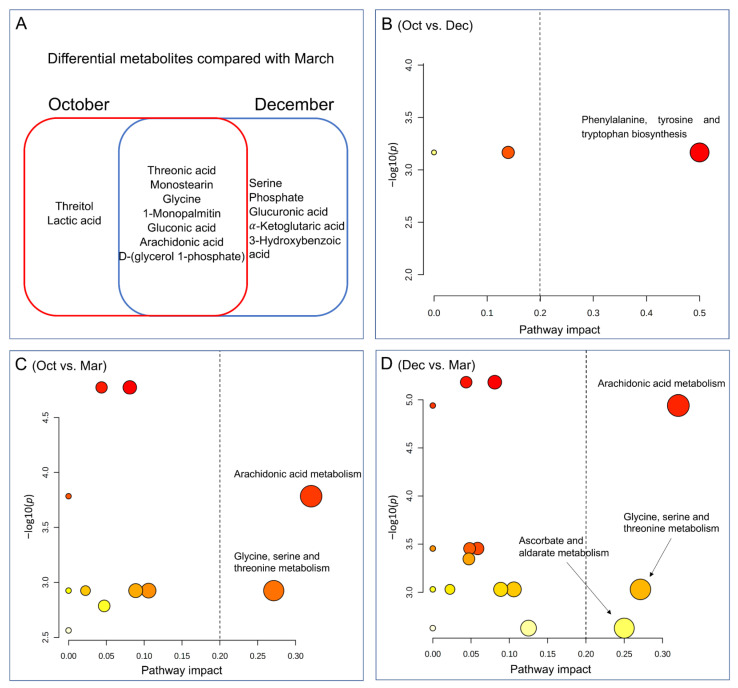
(**A**) summary of the differential serum metabolites in October or December compared with March; pathway impact analysis based on the differential serum metabolites between (**B**) October and December; (**C**) October and March; (**D**) December and March. Pathway impact > 0.20 and false discovery rate (FDR) < 0.05 were deemed as the key enriched pathways.

**Figure 4:**
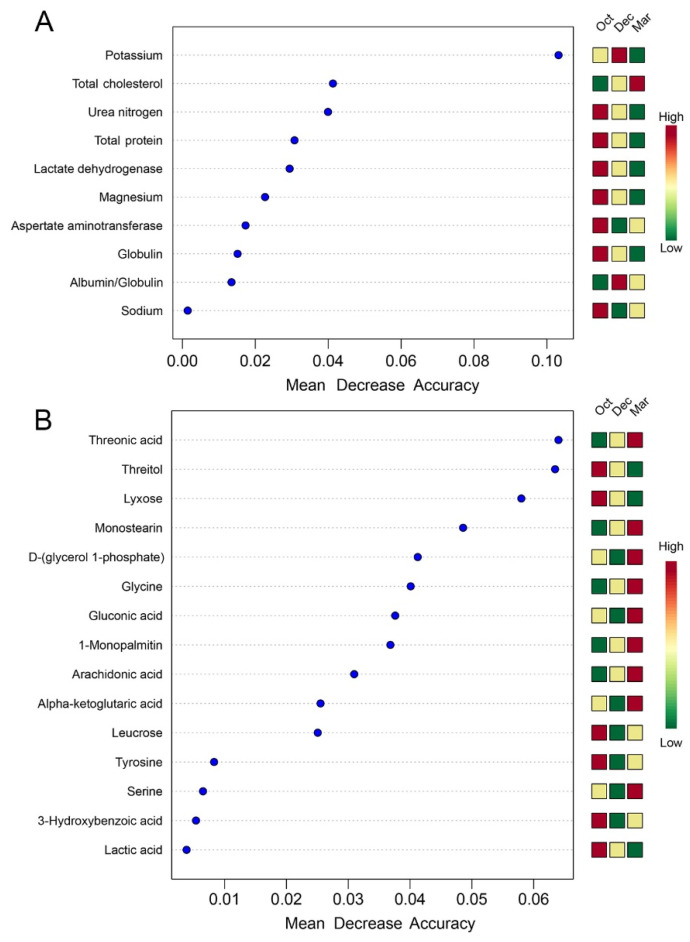
Random forest models of (**A**) serum indices with significant differences and (**B**) differential serum metabolites in grazing yaks during the cold season. Only the indices with a false discovery rate (FDR) < 0.05 are presented in the figures.

The differential metabolites of yak serum were further analyzed by the pathway impact. Figure 3B showed that only the pathway of “phenylalanine, tyrosine, and tryptophan biosynthesis” was enriched between October and December (pathway impact = 0.50, FDR < 0.001). The differential serum metabolites were similar between October to March, and December to March, which shared seven differential metabolites (Figure 3A). The pathways of “arachidonic acid metabolism” (pathway impact = 0.32, FDR < 0.001) and “glycine, serine, and threonine metabolism” (pathway impact = 0.27, FDR = 0.002) were enriched between October and March (Figure 3C), while three pathways were enriched between December and March including “arachidonic acid metabolism” (pathway impact = 0.32, FDR < 0.001), “glycine, serine, and threonine metabolism” (pathway impact = 0.27, FDR = 0.001), and “ascorbate and aldarate metabolism” (pathway impact = 0.25, FDR = 0.002) (Figure 3D). Random forest analysis was conducted to clarify the contribution of serum indices with significant differences and differential serum metabolites (Figure 4). In total, three key serum indices (i.e., potassium, total cholesterol, and urea nitrogen) and six key differential serum metabolites (i.e., threonic acid, threitol, lyxose, monostearin, D-(glycerol 1-phosphate), and glycine) among the grazing yaks during the cold season under the conditions of FDR < 0.01 and mean decrease accuracy ≥ 0.04.

## 4. Discussion

### 4.1. Effects of Dietary Deficiencies on the Blood Metabolic Profiles of Grazing Yaks between October and March during the Cold Season

Due to the lack of grain sources and outdated animal husbandry, grazing is the major feeding pattern and the forages in the meadow are the direct feeds for yaks on the Qinghai-Tibet Plateau [6]. However, the harsh environment on this plateau decreases the production and nutritive levels of forages during the long cold season (October to the following May) [2,41]. In the present experiment, the content of ether extract in the forages collected in March was significantly lower than that of the forages collected in October and December, which was in agreement with Guo et al. [2]. However, the contents of organic matter and fiber materials had no difference among sampling days. The reason could be that the forages in the experiment pasture were highly lignified and low-quality, since more than 65% of their composition was neutral detergent fiber. Thus, the seasonal changes caused minor effects on the nutrient compositions of these forages.

In the present experiment, yaks suffered starvation in March compared with October and December because of the shortage in plant biomasses, which could cause the changes in blood biochemicals and metabolites in response to the deficiency of specific nutrients [25]. Serum biochemicals involved in the protein metabolism showed a larger variation than the carbohydrate metabolism between October and March. Both limited intakes of nitrogen and energy had serious impacts on the rumen microbes of yaks, especially the key role of energy on the synthesis of microbial crude protein [42], which could be supported by the significantly lower concentrations of ruminal ammonia-N and microbial crude protein in March than those in October. The blood urea derives from the ruminal ammonia that is transformed in the liver [43], while the blood protein levels of ruminants are affected by the dietary nitrogen intake [29]. The decrease of ruminal ammonia-N and microbial crude protein in March caused by starvation in yaks would affect their protein metabolism. Zhou et al. reported that blood concentrations of urea, total protein, and albumin in grazing yaks decreased without supplementary feeds [31]. Thus, the limitation of both dietary energy and nitrogen would further result in lower serum concentrations of total protein, globulin, and urea-nitrogen of yaks in March in the present experiment, which was in agreement with Zhou et al. [31].

Dietary energy density is positive in the blood concentration of glucose in cattle [44]. In the present experiment, serum glucose was not altered among different months. The reason could be that specific gut bacteria genera altered with seasons would help yaks to digest efficiently the lignocellulosic materials in winter forages compared with other cattle [2,9], which could provide more precursors for gluconeogenesis in the liver. Besides, the serum concentration of triglyceride in yaks showed no differences among months during the cold season in the present results, which was in agreement with Ding et al. [45]. Previous research reported that serum concentration of total cholesterol in yaks during the cold season was much lower than that during the warm season, suggesting lower serum cholesterol might indicate the shortage of intakes of dry matter and energy in yaks [46,47]. However, in the present experiment, yaks had lower serum concentrations of total cholesterol in October than those in December and March, which was not in agreement with the above results. The reason could be that the nutrient composition of low-quality forages in the pasture changed slightly in the present experiment. In the present experiment, dietary deficiencies resulted in the significant decrease of serum potassium and magnesium, and the decreased tendency of serum calcium and phosphorus in grazing yaks between October and March. Zhou et al. reported that the content of potassium in pasture forages of the Qinghai showed no difference between the withered (December to April of next year) and withering periods (September to November), while magnesium levels were significantly higher in the forages during the withering period [48]. In addition, the increased calcium content and similar phosphorus content were observed in the forages collected in March compared with those collected in October. Thus, shortage of feeds or low absorption of these minerals would be the reason for the decrease of them in grazing yaks during the cold season.

For serum differential metabolites, gluconic acid (gluconate) is a metabolite in the pentose-phosphate pathway. Previous research reported that the plasma gluconate was increased by long-term caloric restriction [49]. In the present experiment, the increased serum gluconic acid in March could be an indicator to identify yaks suffering from starvation or dietary deficiencies of energy and protein in March. Furthermore, monostearin and 1-monopalmitin are monoglycerides and their concentrations are negatively correlated to blood glucose [50]. Thus, the greater concentrations of these two monoglycerides in the serum of yaks collected in March than in October and December could be attributed to the serum glucose among different months. Results also showed that the greater serum arachidonic acid and glycine of yaks in March affected the pathways of “arachidonic acid metabolism” and “glycine, serine, and threonine metabolism” according to the analysis of pathway impact. Arachidonic acid is an essential fatty acid obtained from foods and its concentration in blood is affected by the intake of arachidonic acid [51]. Thus, the different content of arachidonic acid in forages among months could be the reason for the greater serum arachidonic acid of yaks in March. Besides, glycine is a proteinogenic amino acid that is involved in the body protein turnover [52] and conditionally affects the regeneration efficiency of skeletal muscle regeneration in mammals [53]. Serum glycine of grazing yaks increased in March compared with the other two months in the present experiment. The reason could be that dietary energy or protein deficiency caused the muscle breakdown in these yaks and subsequently increased glycine turnover in the body, which was in agreement with the results in cows suffering malnutrition during early lactation [54]. Results of the random forest showed that the concentrations of total protein, urea nitrogen, and glycine were key metabolic profiles in yaks between October and March, suggesting that dietary deficiency of nutrients caused by starvation would be the major detrimental factor for the health status of yaks during the cold season.

### 4.2. Effects of Cold Stress on the Blood Metabolic Profiles of Grazing Yaks between October and December during the Cold Season

Grazing yaks also suffer cold stress under extremely low temperatures in winter. For example, the temperatures in December were much lower than those in October in the present experiment (−4 to 11 °C vs. −20 to 0 °C) with no difference in the plant biomass of pasture for the yaks. Thus, the differences in blood metabolic profiles of yaks between October and December would be attributed to the cold stress mainly. A previous study reported that cold exposure increased the dry matter intake and decreased the apparent digestibility of dry matter, organic matter and nitrogen, as well as the concentration of ruminal ammonia in sheep [55]. These results were in agreement with our results that the concentrations of ruminal ammonia and microbial crude protein in yaks were both lower in December than in October, suggesting a decreased nitrogen digestion of them. Nazifi et al. [4] reported that the cold stress (4 °C) caused the decrease of urea nitrogen, triglyceride, activities of alanine aminotransferase and alkaline phosphatase, and the increase of total protein, glucose, cholesterol, and total lipid in sheep blood compared to those under the optimum temperature (21 °C). In the present experiment, yaks that suffered cold stress in December had lower serum concentrations of total protein, globulin, and activity of aspartate aminotransferase, but greater serum cholesterol than those in October. These results were contradictory to Nazifi et al. [4]. Serum activity of aspartate aminotransferase is positive with the intensity of metabolic changes in the hosts, especially protein metabolism [56]. The acute decrease of feeds for grazing yaks from September to November [2] could cause negative energy balance and muscle damage, and subsequently increase the activities of lactate dehydrogenase and aspartate aminotransferase on 25 October rather than on 25 December or 25 March. However, the actual mechanism still needs to be investigated. Cold stress would also affect the absorption efficiency of minerals in mammals, such as the depressed absorption of calcium in young turkeys [57], which could be the reason for the decreased serum magnesium level and the similar calcium level of grazing yaks in December compared with October of the present experiment, even though the calcium content in forages significantly increased in December.

For the small metabolites, the results identified only five differential metabolites in the serum of yaks between October and December, suggesting yaks would have a stronger adaption to the cold environment than other ruminants due to their long-term evolution. Based on these differential metabolites, the pathway “phenylalanine, tyrosine, and tryptophan biosynthesis” of yaks was greatly inhibited in December compared with that in October, due to the decreased serum concentration of tyrosine in December. Previous research reported that cold stress would raise the activity of tyrosine hydroxylase, which is the rate-limiting enzyme for the synthesis of catecholamines including epinephrine, norepinephrine, and dopamine in different tissues of mammals [58]. Thus, the decreased serum tyrosine and inhibition of the pathway “phenylalanine, tyrosine, and tryptophan biosynthesis” could be attributed to the increased activity of tyrosine hydroxylase in yaks’ tissues caused by cold stress in the present experiment. Tyrosine supplementation would improve the ability of mammals to maintain their core temperature [59]. Though serum metabolites of grazing yaks were slightly changed by the cold stress, supplementary tyrosine could be beneficial for them to resist the harsh environment during the cold season. According to the results of random forest, the key metabolic profiles of yaks’ serum (FDR < 0.05, mean decrease accuracy > 0.04) did not have obvious differences between October and December. This suggests that yaks would tolerate the cold stress from −4~11 °C in October to −20~0 °C in December, which could be attributed to their physiological structure and adaptation to the harsh environment in the highlands. However, the dietary deficiencies of nutrients especially the energy and protein had greater effects on the metabolic profiles in the serum of yaks as well as their metabolic status during the cold season. It is worth noting that the percentage of crude protein in the loss of empty body weight of grazing yaks during the cold season was much higher than that of fat (44.0% vs. 24.3%) [6], suggesting the protein degradation in the body would be more hugely than fat degradation to compensate for the lack of energy during this period. Supplementing energy feeds (barley) would be more useful to prevent the weight loss of grazing yaks during the cold season than supplementing the protein source (rapeseed meal) [31].

## 5. Conclusions

Summarily, dietary nutrient deficiency would be more influential on the physiological status of grazing yaks than the cold stress during the cold season, since the metabolic profiles related to the protein and amino acid metabolism showed great changes in the serum of grazing yaks. The metabolism of protein and amino acids of grazing yaks was affected more greatly by nutrient deficiency than the metabolism of energy and minerals during the cold season based on the analysis of the random forest. Supplementary feeding would be beneficial for improving the metabolic status of grazing yaks during the cold season, but it still needs to be further investigated.

## Figures and Tables

**Table 1:** Nutritional value of forages in different months of cold season (dry matter basis).

Item (% of Dry Matter)	Oct	Dec	Mar	*p*-Value
Organic matter	97.75 ± 0.24	97.73 ± 0.19	97.93 ± 0.04	0.554
Non-fiber carbohydrate *	25.59 ± 0.78 ^ab^	23.65 ± 1.34 ^b^	26.33 ± 0.73 ^a^	0.011
Crude protein	4.48 ± 0.40 ^a^	3.80 ± 0.26 ^b^	4.17 ± 0.28 ^ab^	0.010
Ether extract	2.24 ± 0.19 ^a^	2.42 ± 0.11 ^a^	1.32 ± 0.21 ^b^	0.002
Neutral detergent fiber	65.44 ± 1.66	67.86 ± 3.96	66.11 ± 2.74	0.609
Acid detergent fiber	36.63 ± 0.58	37.18 ± 1.13	37.01 ± 0.61	0.707
Calcium	0.376 ± 0.047 ^a^	0.817 ± 0.022 ^b^	0.862 ± 0.038 ^b^	<0.001
Phosphorus	0.026 ± 0.001 ^a^	0.017 ± 0.001 ^b^	0.024 ± 0.006 ^a^	0.035

Values with different superscripts represented significant differences among groups (*p* < 0.05). Results were presented as mean ± standard deviation. * Contents of non-fiber carbohydrates in forages were calculated by the contents of dry matter minus the percentages of crude protein, ash, ether extract, and neutral detergent fiber in forages.

**Table 2:** Dynamic changes in serum biochemical indices of grazing yaks during cold season.

Items	Oct	Dec	Mar	SEM	*p*-Value
Protein metabolism					
Total Protein (g/L)	78.4 ^a^	69.8 ^b^	64.8 ^b^	1.82	<0.001
Albumin (g/L)	32.9 ^ab^	34.2 ^a^	30.5 ^b^	0.96	0.044
Globulin (g/L)	45.5 ^a^	35.6 ^b^	34.3 ^b^	1.86	0.001
Albumin/Globulin	0.735 ^b^	0.972 ^a^	0.895 ^ab^	0.049	0.012
Urea nitrogen (mmol/L)	7.06 ^a^	6.04 ^a^	3.56 ^b^	0.553	0.002
Energy metabolism					
Glucose (mmol/L)	4.58	4.53	3.96	0.223	0.130
Total cholesterol (mmol/L)	1.94 ^b^	2.53 ^a^	2.79 ^a^	0.111	<0.001
Triglyceride (mmol/L)	0.247	0.350	0.363	0.055	0.291
Metabolic enzymes					
Alanine transaminase (μkat/L)	0.488	0.653	0.528	0.053	0.104
Aspartate aminotransferase (μkat/L)	1.40 ^a^	1.07 ^b^	1.10 ^b^	0.068	0.007
Alkaline phosphatase (μkat/L)	2.34	1.84	1.38	0.256	0.057
Lactate dehydrogenase (μkat/L)	16.8 ^a^	14.4 ^ab^	12.4 ^b^	0.65	0.001
Minerals utilization					
Potassium (mmol/L)	5.06 ^a^	5.24 ^a^	4.50 ^b^	0.098	<0.001
Sodium (mmol/L)	142.3 ^a^	138.7 ^b^	140.0 ^ab^	0.91	0.038
Calcium (mmol/L)	2.48	2.41	2.26	0.065	0.088
Magnesium (mmol/L)	0.987 ^a^	0.770 ^b^	0.745 ^b^	0.047	0.005
Iron (μmol/L)	26.2	20.8	21.6	2.09	0.182
Phosphorus (mmol/L)	2.88	2.02	2.09	0.227	0.053

Values with different superscripts represent significant differences among groups (*p* < 0.05).

**Table 3:** Differential serum metabolites in yaks grazing in different months of the cold season.

Metabolite Name	Retention Time (min)	Mass	Similarity	VIP ^a^	Fold Change ^b^	FDR ^c^
Oct vs. Dec						
Lyxose	10.76	217	631.8	1.94	1.358	0.019
Threonic acid	10.19	292	888.6	1.97	0.652	0.019
Threitol	9.87	217	873.6	1.91	1.527	0.027
Tyrosine	12.39	218	950.9	1.97	1.523	0.027
Leucrose	16.80	73	506.8	1.91	2.197	0.035
Oct vs. Mar						
Monostearin	16.57	57	581.8	2.21	0.265	<0.001
1-Monopalmitin	15.40	57	751.8	2.13	0.363	<0.001
Threonic acid	10.19	292	888.6	2.11	0.522	<0.001
D-(glycerol 1-phosphate)	11.39	299	793.4	2.06	0.385	0.001
Threitol	9.87	217	873.6	2.02	1.596	0.003
Arachidonic acid	14.42	91	737.3	1.98	0.332	0.004
Glycine	7.23	102	819.3	1.86	0.261	0.027
Gluconic acid	12.66	292	602.4	1.83	0.406	0.033
Lactic acid	6.77	59	806.5	1.79	1.473	0.048
Dec vs. Mar						
Monostearin	16.57	57	581.8	2.37	0.278	<0.001
1-Monopalmitin	15.40	57	751.8	2.30	0.487	<0.001
D-(glycerol 1-phosphate)	11.39	299	793.4	2.34	0.339	<0.001
Arachidonic acid	14.42	91	737.3	2.31	0.421	<0.001
α-Ketoglutaric acid	10.33	198	845.4	2.08	0.514	0.011
Gluconic acid	12.66	292	602.4	2.08	0.346	0.012
Serine	8.93	204	934.1	2.10	0.525	0.015
Glycine	7.23	102	819.3	2.02	0.488	0.019
Phosphate	8.33	158	818.8	1.98	0.591	0.023
3-Hydroxybenzoic acid	10.35	120	546.4	1.95	0.843	0.033
Glucuronic acid	12.43	333	479.6	1.92	0.865	0.034
Threonic acid	10.19	292	888.6	1.90	0.800	0.047

^a^ Variable important in projection (VIP) was obtained from the orthogonal partial least squares discriminant analysis (PLS-DA) of serum metabolites in yaks. ^b^ Fold changes were calculated by the area of each metabolite between groups (former month/later month). ^c^ FDR represents the false discovery rate.

## Data Availability

Data that are reported in the present experiment were not deposited in an official repository, but are available from the authors upon request.

## Abbreviations

PCA: Principal component analysis; PLS-DA, partial least squares-discriminant analysis; VIP, Variable important in projection; FDR, false discovery rate; GC-TOF/MS, gas chromatograph coupled with time-of-flight mass spectrometer.
